# Supplementary material for: Bacterial microcompartments for isethionate desulfonation in the taurine-degrading human-gut bacterium Bilophila wadsworthia
Source: BMC Microbiol. 2021 Dec 13;21:340. doi: 10.1186/s12866-021-02386-w (PMC8667426; doi:10.1186/s12866-021-02386-w)
Supplement: Supplementary file 1 — Additional file 1. [file 12866_2021_2386_MOESM1_ESM.docx]

**Supplemental information
for manuscript:**

**Bacterial microcompartments for isethionate desulfonation
in the taurine-degrading human-gut bacterium *Bilophila wadsworthia***

Anna G. Burrichter^a,b^#, Stefanie Dörr^a^, Paavo Bergmann^c^, Sebastian Haiß^a^, Anja Keller^a,b^, Corentin Fournier^a,d^, Paolo Franchini^a^, Erika Isono^a^, David Schleheck^a,b^ #

^a^Department of Biology, University of Konstanz, Konstanz, Germany
^b^Konstanz Research School Chemical Biology, University of Konstanz, Konstanz, Germany
^c^Electron Microscopy Centre, Department of Biology, University of Konstanz, Konstanz, Germany

#Correspondence: Anna Burrichter, anna.burrichter@uni-konstanz.de and David Schleheck, david.schleheck@uni-konstanz.de


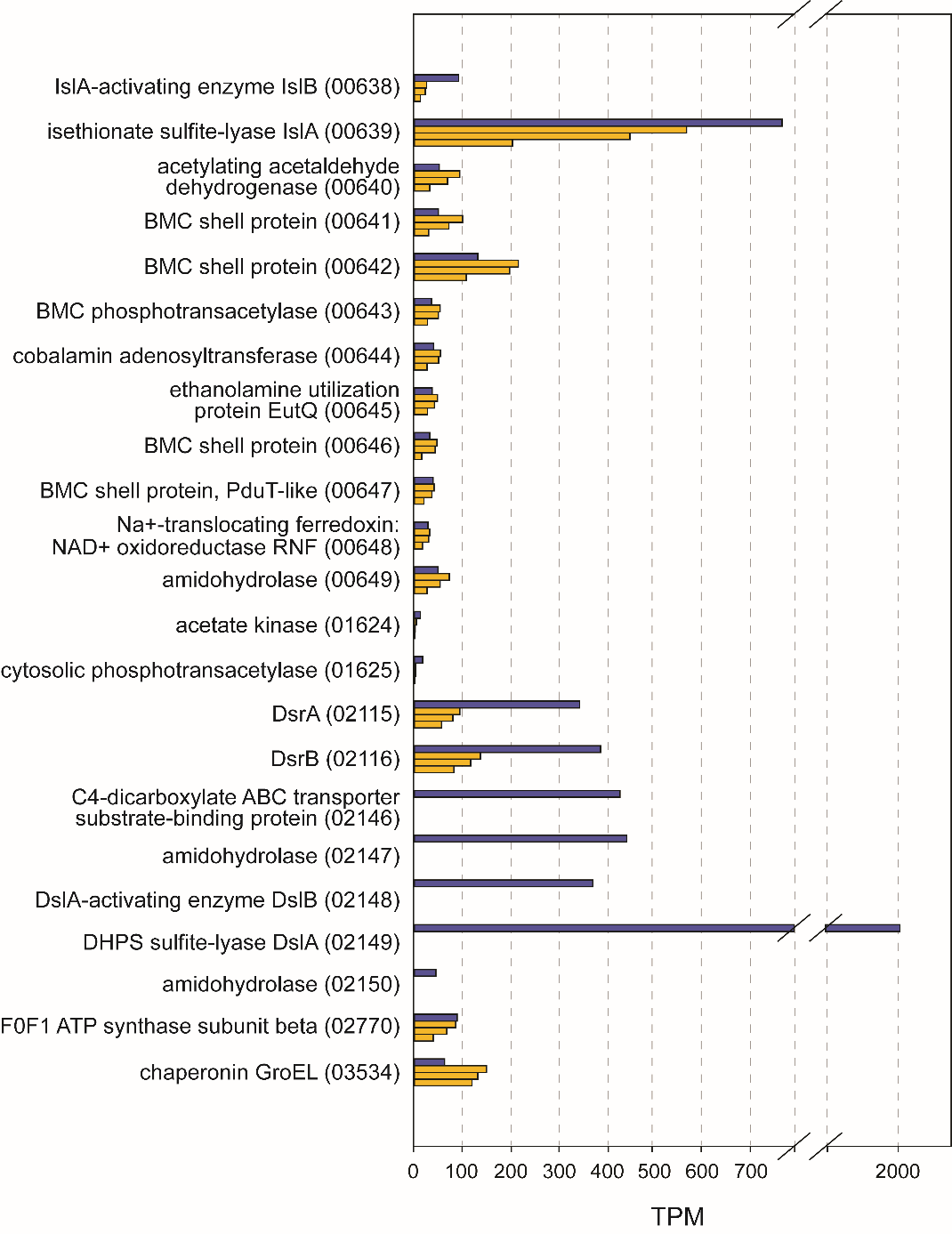


**Figure S1: Transcriptomic data for genes relevant to the discussed pathways.**

Data is shown as transcripts per million reads. Blue bars denote transcriptomic data from a culture grown with 20 mM each of lactate and dihydroxypropanesulfonate, yellow bars from cultures grown with 20 mM each of lactate and taurine (three biological replicates). While taurine is metabolized *via* IslAB (locus tags HMPREF0179_00638 and _00639), DHPS is cleaved by a different enzyme, DslAB (HMPREF0179_02148 and _02149) (i.e., 2,3‑dihydroxypropanesulfonate sulfite lyase termed HpsG as most recently described by Liu et al. (1)). Residual expression of taurine-metabolizing genes in the DHPS-grown culture is visible, leading to the assumption that these enzymes are downregulated only slowly when taurine is not present.
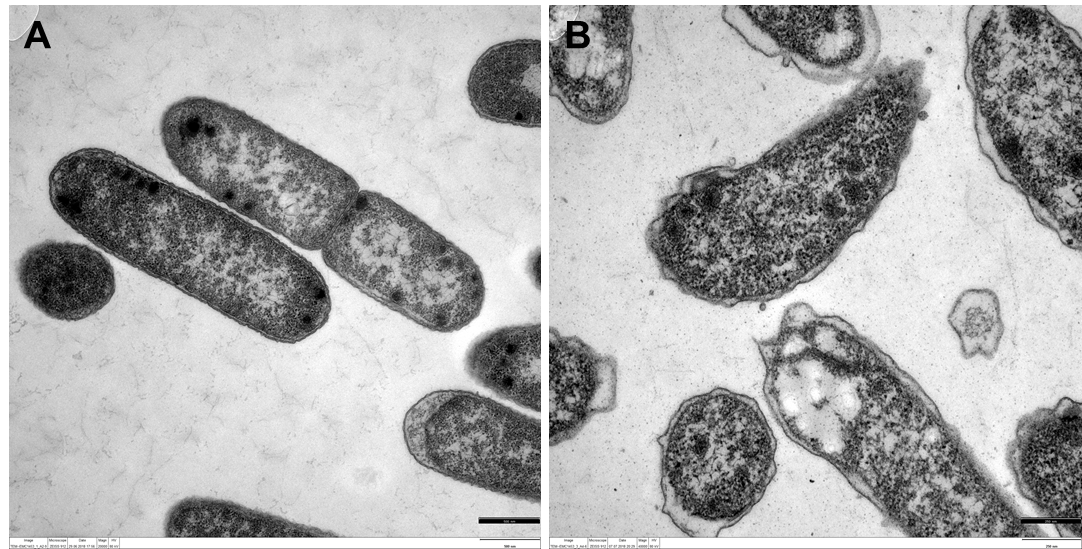


**Figure S2:** **TEM images of ultrathin sections of *B. wadsworthia* cells grown with taurine (A) or *Desulfovibrio alaskensis* cells grown with choline (B) as the electron acceptor (B).**

Polyhedral structures characteristic of BMCs were observed under both conditions. *D. alaskensis* has previously been described to form microcompartments when degrading choline (2). Scale bars: 500 nm.


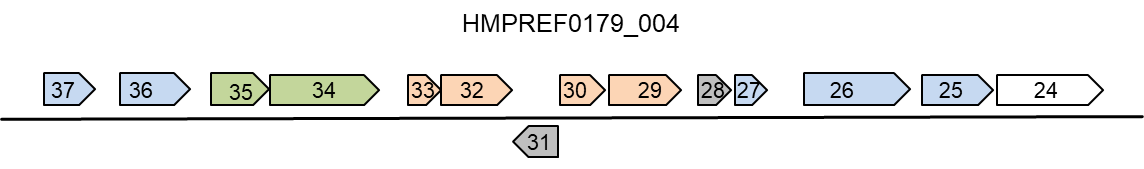


Figure S3: Illustration of the gene cluster in *B. wadsworthia* encoding sulfolactate degradation enzymes including sulfolactate sulfo-lyase (SuyAB).

Genes marked in orange are directly involved in sulfolactate degradation: 00433, SuyA; 00432, SuyB; 00430, (S)-sulfolactate dehydrogenase; 00429, (R)-sulfolactate dehydrogenase. The two dehydrogenases most likely interconvert sulfolactate enantiomers, analogous to the pathway described by (3). Genes marked in green encode transport components: 00435, tripartite-type tricarboxylate transporter, receptor component TctC; 00434, putative tricarboxylic transport membrane protein. Genes marked in blue are annotated to encode metabolic enzymes but their function related to sulfolactate metabolism is not clear: 00437, hydroxyethylthiazole kinase (from thiamine metabolism); 00436, Formate dehydrogenase maturation protein FdhE; 00427, methylglyoxal synthase; 00426**,** D-lactate dehydrogenase; 00425**,** glycolate oxidase iron-sulfur subunit. Genes marked in grey may serve a regulatory function: 00431**,** GntR family transcriptional regulator; 00425**,** regulator of RNase E activity RraA. 00424 annotated to encode a hypothetical protein. Notably, there are no genes found in the gene cluster that are annotated to encode BMC shell proteins. All annotations were taken from IMG for *Bilophila wadsworthia 3.1.6*. and the shortened IMG locus tag can be completed by adding the given number to the following prefix: HMPREF0179_.

Table S1: Number of reads from transcriptomic analysis for RNA samples from
*B. wadsworthia* grown with lactate/taurine or lactate/dihydroxypropanesulfonate (DHPS)

|  |  | Taurine_1 | Taurine_2 | Taurine_3 | DHPS |
| --- | --- | --- | --- | --- | --- |
| Raw reads | | 25,780,974 | 21,425,313 | 12,520,332 | 14,901,949 |
| Filtered reads  (% of total raw reads) | | 25,692,021 (99.65%) | 21,347,365 (99.64%) | 12,473,023 (99.62%) | 14,854,536 (99.68%) |
| reads aligned to reference genome (alignment rate) | | 25,604,277 99.66 % | 21,157,452 99.11 % | 12,430,468 99.66 % | 14,808,153 99.69 % |

Table S2: Proteomic results for the SDS-PAGE bands (see Figure 4C). The protein description includes a shortened locus tag, which can be completed by adding the shown number to the following prefix: HMPREF0179_0. BMC shell proteins are marked bold.

| Fraction | approximate protein size | identified as | Score |
| --- | --- | --- | --- |
| 3 | 10 kDa (1) | **ethanolamine utilization protein EutN (0642)** | 115 (top score) |
|  | 10 kDa (2) | integration host factor subunit alpha (1532) | 242.03 |
|  |  | taurine-pyruvate aminotransferase (2713) | 225.79 |
|  |  | chaperonin GroES (3535) | 196.43 |
|  |  | DNA-binding protein HU-beta (0718) | 191.42 |
|  |  | hypothetical protein (0544) | 188.06 |
|  |  | **ethanolamine utilization protein EutN (0642)** | 157.39 |
|  | 25 kDa | dissimilatory sulfite reductase beta subunit (2116) | 655.11 |
| 4 | 14 kDa | LSU ribosomal protein L10P (2084) | 227.30 |
|  | 15 kDa | LSU ribosomal protein L10P (2084) | 287.38 |
|  |  | periplasmic chaperone for outer membrane proteins Skp (2805) | 234.45 |
|  |  | SSU ribosomal protein S5P (2023) | 234.24 |
|  |  | LSU ribosomal protein L9P (2745) | 193.54 |
|  |  | Class III cytochrome C family protein (1235) | 189.18 |
|  |  | small subunit ribosomal protein S9 (0867) | 187.27 |
|  |  | SSU ribosomal protein S7P (2067) | 161.72 |
|  |  | hypothetical protein (2302) | 151.57 |
|  |  | Flavorubredoxin (3149) | 140.29 |
|  |  | **Carboxysome shell and ethanolamine utilization microcompartment protein CcmL/EutN (0647)** | 117.07 |
|  | 18 kDa | ATP-dependent Clp protease, protease subunit (2214) | 138.21 |
|  |  | transcriptional regulator, TetR family (0863) | 135.34 |
|  |  | hypothetical protein (0984) | 134.65 |
|  |  | LSU ribosomal protein L9P (2745) | 130.60 |
|  |  | pyrimidine operon attenuation protein / uracil phosphoribosyltransferase (3139) | 119.28 |
|  |  | Rubrerythrin (2466) | 95.03 |
|  |  | Putative exonuclease, RdgC (1649) | 87.47 |
|  |  | bacterioferritin (0484) | 82.44 |
|  |  | **Carboxysome shell and ethanolamine utilization microcompartment protein CcmL/EutN (0647)** | 81.44 |

**Table S3: Coomassie and silver staining of SDS polyacrylamide gels, modified after (4, 5).**

|  | Substance | time |
| --- | --- | --- |
| Coomassie staining | 0.25 % (w/v) Coomassie brilliant blue R-250, 45% (v/v) ethanol , 10% (v/v) acetic acid in water | 1 h |
| Washing/initial destaining | 45% (v/v) ethanol , 10% (v/v) acetic acid in water | 1 h |
| Destaining/Fixation | 45% (v/v) ethanol , 10% (v/v) acetic acid in water | 14 h |
| Reduction | 30% (v/v) ethanol, 0.8 M acetate, 2 g/l thiosulfate | 30 min |
| Washing | distilled water | 3x 5 min |
| Silver solution | 2 g/l (11.8 mM) silver nitrate, 200 µl/l 37 % formaldehyde | 20 min |
| Developing | 25 g/l (0.236 M) sodium carbonate, 100 µl/l 37 % formaldehyde | 2-8 min |
| Stopping | 10 g/l glycine | 10 min |

Table S4: Cell fixation and embedding protocol for transmission electron microscopy

|  | Substance | pH | °C | time |
| --- | --- | --- | --- | --- |
| Pre fixation | 50/50 culture medium with 5% glutardialdehyde in 0.05M HEPES | 7 | 0 | 45 min |
| Enclosing | Agarose-enclosure | | | |
| Fixation | 2.5% glutardialdehyde in 0,1M HEPES | 7 | 0-4 | 2.5 h |
| Washing | 0.05M HEPES | 7 | 0 | 3x 10 min |
| Postfixation / osmification | 2% OsO_4_ in 0.05M HEPES | 7 | 0 | 1h |
| Washing | 0.05M HEPES | 7 | 0 | 3x 10 min |
| Drainage | 30% ethanol, precooled |  | 4 | 10 min |
|  | 50% ethanol, precooled |  | 4 | 15 min |
| En-bloc staining | Uranylacetat saturated in 70% ethanol |  | 4 | o.n. |
| Dehydration | 70% ethanol, precooled |  | RT | 3x 10 min |
|  | 80% acetone, precooled |  | RT | 3x 10 min |
|  | 90% acetone, precooled |  | RT | 3x 10 min |
|  | 96% acetone, precooled |  | RT | 3x 10 min |
|  | 100% acetone, dried on molecular sieve |  | RT | 3x 10 min |
| Intermedium | 100% acetone, dried on molecular sieve |  | RT | 1 h |
| Embedding | 15% Spurr resin in acetone |  | RT | 1 h |
|  | 33% Spurr resin in acetone |  | RT | 2 h |
|  | 50% Spurr resin in acetone |  | RT | 2 h |
|  | 75% Spurr resin in acetone |  | RT | o.n. |
|  | pure Spurr resin in closed Eppies |  | RT | 2x 2 h |
| Polymerisation |  |  | 65 | 48 h |
| Cooling | In extractor hood |  | RT | overnight |

# Supplemental references

1. Liu J, Wei Y, Lin L, Teng L, Yin J, Lu Q, Chen J, Zheng Y, Li Y, Xu R, Zhai W, Liu Y, Liu Y, Cao P, Ang EL, Zhao H, Yuchi Z, Zhang Y. 2020. Two radical-dependent mechanisms for anaerobic degradation of the globally abundant organosulfur compound dihydroxypropanesulfonate. Proc Natl Acad Sci U S A 117:15599-15608.

2. Kuehl JV, Price MN, Ray J, Wetmore KM, Esquivel Z, Kazakov AE, Nguyen M, Kuehn R, Davis RW, Hazen TC, Arkin AP, Deutschbauer A. 2014. Functional genomics with a comprehensive library of transposon mutants for the sulfate-reducing bacterium *Desulfovibrio alaskensis* G20. mBio 5:e01041-14.

3. Mayer J, Huhn T, Habeck M, Denger K, Hollemeyer K, Cook AM. 2010. 2,3-Dihydroxypropane-1-sulfonate degraded by *Cupriavidus pinatubonensis* JMP134: purification of dihydroxypropanesulfonate 3-dehydrogenase. Microbiology 156:1556-64.

4. Heukeshoven J, Dernick R. 1985. Simplified method for silver staining of proteins in polyacrylamide gels and the mechanism of silver staining. Electrophoresis 6:103-112.

5. Laemmli UK. 1970. Cleavage of structural proteins during the assembly of the head of bacteriophage T4. Nature 227:680-685.
